# Association between hashimoto thyroiditis and differentiated thyroid cancer: A single-center experience

**DOI:** 10.3389/fonc.2022.959595

**Published:** 2022-07-28

**Authors:** Federico Cappellacci, Gian Luigi Canu, Maria Letizia Lai, Eleonora Lori, Miriam Biancu, Francesco Boi, Fabio Medas

**Affiliations:** ^1^ Department of Surgical Sciences, University of Cagliari, Cagliari, Italy; ^2^ Department of Cytomorphology, University of Cagliari, Cagliari, Italy; ^3^ Department of Surgical Science, Sapienza University of Rome, Rome, Italy; ^4^ Endocrinology, Department of Medical Sciences and Public Health, University of Cagliari, Cagliari, Italy

**Keywords:** hashimoto thyroiditis, differentiated thyroid cancer, thyroid surgery, papillary thyroid cancer, thyroid

## Abstract

Hashimoto’s thyroiditis is the most common cause of hypothyroidism in the iodine-sufficient areas of the world. Differentiated thyroid cancer is the most common thyroid cancer subtype, accounting for more than 95% of cases, and it is considered a tumor with a good prognosis, although a certain number of patients experience a poor clinical outcome. Hashimoto’s thyroiditis has been found to coexist with differentiated thyroid cancer in surgical specimens, but the relationship between these two entities has not yet been clarified. Our study aims to analyze the relationship between these two diseases, highlighting the incidence of histological diagnosis of Hashimoto thyroiditis in differentiated thyroid cancer patients, and assess how this autoimmune disorder influences the risk of structural disease recurrence and recurrence rate.

## Introduction

Hashimoto’s thyroiditis (HT) is the most common autoimmune inflammatory pathology of the thyroid and is the leading cause of hypothyroidism in iodine-sufficient areas of the world. It is characterized by a lymphocytic infiltrate, in particular T-cells, able to determine the follicular destruction of the gland, its fibrous involution, and consequent hypothyroidism (1–5).

Thyroid cancer is the fifth most common cancer in the USA, and over 44 000 new cases occurred in men and women in 2021. The incidence of thyroid cancer is still rising worldwide, mostly due to the increased use of diagnostic imaging and surveillance, and the diffusion of more accurate cytological classifications (6–12). Differentiated thyroid cancer (DTC) is the most common subtype, accounting for more than 95% of cases, and it is generally considered a tumor with a good prognosis, although a certain number of patients experience a poor clinical outcome (6, 8, 13–15).

Hashimoto’s thyroiditis has been found to coexist with DTC in surgical specimens, but the relationship between these two entities has not yet been clarified in the literature (3, 16–25). Many authors suggest that patients with a diagnosis of HT, especially those with the nodular variant, should undergo a strict follow-up, as DTC seems to be more frequently encountered in those cases. Many studies report the frequent coexistence of these two pathologies in pathological examinations, but the clinical mechanisms linking these two pathologies and whether HT could be a protective or promoting factor for DTC progression remain unclear, as some authors report a higher frequency of multifocality in DTC with concomitant HT, while others suggest that the coexistence of HT in DTC patients is associated with favorable clinical outcomes (3, 17, 20–22).

Our study aims to analyze the relationship between HT and DTC, assessing the incidence of HT in DTC patients, and to evaluate whether HT influences the risk of structural disease recurrence, as described in 2015 ATA guidelines (26), and the disease-free survival rate in patients with DTC.

## Methods

### Study design

This was a retrospective cohort study that included patients who underwent thyroid surgery between January 2016 and December 2019 in our Unit of General and Endocrine Surgery. Patients were identified from a prospectively maintained institutional database. Data from 1981 consecutive patients were screened. Patients with a histological diagnosis of medullary or anaplastic thyroid cancer, those with a preoperative diagnosis of Graves’ disease or toxic goiter, or those with incomplete data were excluded from this study. A total of 839 patients met the inclusion criteria and were enrolled in this study.

First, we assessed the incidence of HT in patients with a pathological diagnosis of benign or malignant thyroid disease.

Histological diagnosis of HT was made based on the presence in surgical specimens of the following characteristics: diffuse lymphoplasmacytic infiltration, germinal centers, and enlarged epithelial cells with large nuclei and eosinophilic cytoplasm (Askanazy or Hürthlecells). Non-specific lymphocytic thyroiditis occurring immediately adjacent to a tumor could not be differentiated from perineoplastic inflammation and was not considered HT.

Then, we focused our attention on patients with DTC. Particularly, we divided the patients into two groups according to the presence (HT-DTC group) or absence (non-HT-DTC group) of HT, with the aim of assessing the influence of HT on the risk of structural disease recurrence, as described in 2015 ATA guidelines (26), vascular invasion, multifocality (defined as the presence of two or more tumor sites), tumor size, gross and microscopic extrathyroidal invasion, microcarcinomas (defined as tumors < 1cm in larger diameter), nodal metastasis (calculated as more than 5 lymph node per patient), and type of DTC (Papillary Thyroid Cancer (PCT), aggressive variant of PTC [i.e. tall cell, hobnail variant, columnar cell) and Follicular Thyroid Cancer (FTC)].

### Endpoints

The primary endpoint was to evaluate the incidence of HT in patients with benign or malignant thyroid disease to evaluate whether HT is an independent risk factor for DTC; the secondary endpoint was to assess whether HT is associated with aggressive forms of DTC through the analysis of pathological diagnosis, the ATA class risk of disease recurrence and the incidence of disease recurrence.

### Postoperative management and follow-up

All patients were referred to endocrinologists for postoperative management, from which we obtained information regarding post-operative radioactive iodine (RAI) therapy administration, number of RAI therapy cycles, evidence of recurrence and type of recurrence (local and/or nodal).

Disease-free status was defined as a No Evidence of Disease (NED) and included the following features: no clinical evidence of tumor, no imaging evidence of disease by RAI imaging and/or neck ultrasound, and low serum thyroglobulin (Tg) levels during TSH suppression (Tg < 0.2 ng/mL) or after stimulation (Tg < 1 ng/mL) in the absence of interfering antibodies.

### Statistical analysis

Univariate analysis was conducted using the chi-square test, or Fisher exact test when appropriate, for categorical variables, and Student’s t-test for continuous variables. Factors with a p-value ≤0.10 in univariate analysis were considered potentially significant and were included in the multivariate analysis. Logistic regression analysis was used to identify independent risk factors for developing DTC; the results are presented as odds ratios (OR) with 95% confidence intervals (CIs). The results were considered statistically significant for p-value <0.05. Disease-free survival was defined as the time from initial surgery to the detection of recurrence; the log-rank test was used to estimate the differences in Kaplan-Meier curves for independent risk factors. Calculations were performed with MedCalc R vers. 19.1.3. Continuous variables are reported as the mean ± standard deviation of the mean.

## Results

Among the 839 patients included in our study, 229 (27.3%) were males, with an M/F rate of 1/3. The mean age was 52.1 years (SD 14.9, range 13-87 years). A total of 399 (47.6%) patients were included in the DTC group and 440 (52.4%) were included in the non-DTC group. The characteristics of the sample, including mean age, male sex, presence of HT, and preoperative administration of levothyroxine therapy for hypothyroidism are summarized in **Table 1**. In the univariate analysis, HT was significantly associated with DTC: its incidence was 50.1% in patients with malignant disease, and 38.6% in patients with benign disease (p < 0.001). Moreover, DTC patients were significantly younger than non-DTC patients: in fact, the mean age in the DTC group was 49.5 ± 15 years vs 54.4 ± 14.6 years in the non-DTC group (p < 0.001). No difference in terms of sex or preoperative administration of levothyroxine therapy for hypothyroidism was found.

**Table 1:** Univariate analysis between DTC and non-DTC patients.

	Non-DTC (Benign, 440)	DTC (Malign, 399)	*p*
*Mean Age*	54.4 ± 14.6	49.5 ± 15	**< 0.001**
*Sex male*	116 (24.4%)	113 (28.3%)	0.5769
*Concomitant HT*	170 (38.6%)	200 (50.1%)	**< 0.001**
*Levothyroxine Therapy*	45 (10.2%)	40 (10%)	0.9859

DTC, Differentiated Thyroid Cancer HT, Hashimoto Thyroiditis. Bold values simply indicate a statistical significative p value.

In the multivariate analysis, both age (OR = 0.9805, 95% CI 0.9712 to 0.9899, p < 0.001) and HT (OR = 1.7784, 95% CI 1.3332 to 2.3722, p < 0.001) were found to be independent risk factors for developing DTC. Data from the multivariate analysis are reported in **Table 2**.

**Table 2:** Multivariable analysis between DTC and non-DTC group.

Variable	Coefficient	OR	95% CI	*p*
*Age*	-0,019685	0,9805	0,9712 to 0,9899	**<0,0001**
*Male Sex*	0,27206	1,3127	0,9539 to 1,8063	0,0948
*Levothyroxine Therapy*	-0,23410	0,7913	0,5169 to 1,2113	0,2812
*HT*	0,57570	1,7784	1,3332 to 2,3722	**0,0001**

Significance level p < 0,0001 AUC 0.608. DTC, Differentiated Thyroid Cancer. HT, Hashimoto Thyroiditis. OR, Odds Ratio. CI, Confidence Intervals). Bold values simply indicate a statistical significative p value.

As already described in the methods, we performed a subsequent analysis on patients with DTC. Full data are reported in **Table 3**. The mean age in the HT-DTC group was 48.9 ± 14.8 years, while in the non-HT-DTC group was 50.1 ± 15 years (p=0.4131). There were 38 (19%) male patients in the HT-DTC group and 75 (37.7%) in non-HT-DTC group (p < 0.0001). Significant differences were found in terms of multifocality (40.5% in HT-DTC group, vs 29.6% in non-HT-DTC group; p=0.02), tumor size (13.7 ± 11.9 mm vs 17.6 ± 16.5 mm respectively, p=0.007) and microcarcinomas (44% vs 34.2% respectively, p=0.05). No differences in extrathyroidal extension, vascular invasion, metastatic lymph nodes, or type of DTC were found between the HT-DTC and non-HT-DTC groups.

**Table 3:** Differentiated thyroid cancer patients.

	DTC (399)	HT-DTC (200)	Non-HT-DTC (199)	*p*
*Age (years)*	49.5 ± 14.9	48.9 ± 14.8	50.1 ± 15	0.4131
*Male Sex*	113 (28.3%)	38 (19%)	75 (37.7%)	**< 0.0001**
*Gross extrathyroidal extension*	28 (7%)	12 (6%)	16 (8%)	0.4410
*Microscopic extrathyroidal extension*	20 (5%)	8 (4%)	12 (6%)	0.3703
*Multifocality*	140 (35.1%)	81 (40,5%)	59 (29.6%)	**0.0233**
*Vascular invasion*	19 (4.8%)	7 (3.5%)	12 (6%)	0.2509
*Tumor size (mm)*	15.6 ± 14.5	13.7 ± 11.9	17.6 ± 16.5	**0.0074**
*Microcarcinoma (tumor size < 10 mm)*	156 (39.1%)	88 (44%)	68 (34.2%)	**0.05**
*Patients with 5 or more metastatic lymph node*	17 (4.3%)	10 (5%)	7 (3.5%)	0.6214
*PTC*	220 (55.1%)	117 (58.5%)	103 (51.8%)	0.0691
*Aggressive variants of PTC*	90 (22.6%)	48 (24%)	42 (21.1%)	
*FTC*	89 (22.3%)	35 (17.5%)	54 (27.1%)	

DTC, Differentiated Thyroid Cancer. HT, Hashimoto Thyroiditis. PTC, Papillary Thyroid Cancer. FTC, Follicular Thyroid Cancer. Bold values simply indicate a statistical significative p value.

After stratifying the HT-DTC and non-HT-DTC groups according to the ATA risk of structural disease recurrence, 125 and 120 patients were listed as low risk, 57 and 60 patients as intermediate risk, and 18 and 19 patients as high risk, respectively. No statistically significant difference was found (p=0.9033). The results are shown in **Table 4**.

**Table 4:** Risk of Structural Disease Recurrence between differentiated thyroid cancer with concomitant Hashimoto thyroiditis (HT-DTC group) and differentiated thyroid cancer without concomitant Hashimoto thyroiditis (non-HT-DTC group).

	Low Risk	Intermediate Risk	High Risk	p
**HT-DTC**	125	57	18	0.9033
**Non-HT-DTC**	120	60	19	

DTC, Differentiated Thyroid Cancer; HT, Hashimoto Thyroiditis.

**Figure 1:**
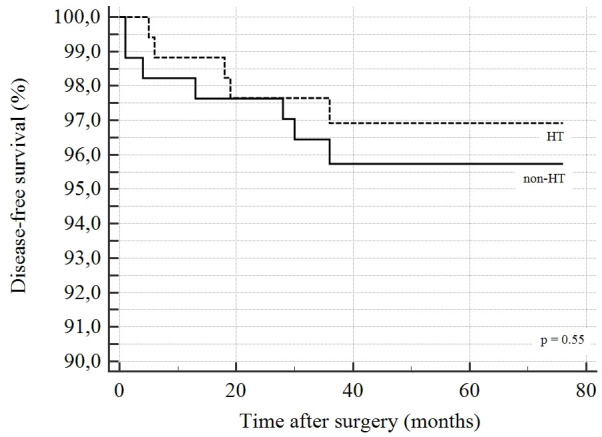
Kaplan-Meier curves estimating disease-free survival in HT-DTC and non-HT-DTC.

Follow-up results of DTC patients are reported in **Table 5**. Sixty patients (15%) were lost to follow-up. The mean follow-up was 50.5 ± 13.8 months (median 50, range 14-76). RAI therapy was administrated to 225 (66.4%) patients. Overall, 12 patients experienced recurrent disease (3.0%). Three patients had local recurrences and 6 patients had nodal recurrences. The mean time between surgery and diagnosis of recurrence was 16.4 ± 13.4 months; the overall survival was 99.5%; only 2 patients died due to DTC. A statistically significant difference was observed in terms of the administration of RAI therapy between the HT-DTC group, which was given to 109 (63.7%) patients vs 116 (69%) patients in the non-HT-DTC group (p=0.04). We found no difference between HT-DTC and non-HT-DTC patients regarding recurrence rates (2.5% vs 3.5%, respectively; p=0.5746), site of recurrence and survival rate. Kaplan-Meier curves for independent factors are reported in **Figure 1**. The log-rank test in Kaplan-Meier curves did not show any significant difference between the HT-DTC and non-HT-DTC groups (p=0.5548).

**Table 5:** Differentiated thyroid cancer follow-up.

Variable	DTC (399)	HT-DTC (200)	Non-HT-DTC (199)	*p*
*FU losses (%)*	60 (15%)	29 (14.5%)	31 (15.6%)	0.8720
*Mean duration of FU (months)*	50.5 ± 13.8	49.5 ± 13.8	51.5 ± 13.8	0.1913
*RAI therapy*	225 (66.4%)	109 (63.7%)	116 (69%)	**0.04**
*RAI therapy cycles (median)*	1	1	1	1
*Disease recurrence*	12 (3.5%)	5 (2.9%)	7 (4.2%)	0.5746
*Mean time from operation to recurrence diagnosis (month)*	16.4 ± 13.4	16.8 ± 12.6	16.1 ± 15	0.9379
*Local Recurrence*	3 (0.9%)	2 (1.2%)	1 (0.6%)	1
*Nodal Recurrence*	6 (1.8%)	2 (1.2%)	4 (2.4%)	0.4489
*Death due to DTC*	2 (0.5%)	1 (0.5%)	1 (0.5%)	1

DTC, Differentiated Thyroid Cancer. HT, Hashimoto Thyroiditis. RAI, Radio-Active Iodine. FU, Follow-up. Bold values simply indicate a statistical significative p value.

## Discussion

Since its first observation in 1955 by Dailey et al., the link between HT and DTC remains a controversial subject. Despite many studies in the literature having exploited this topic, there is not unanimous scientific opinion, and the debate is still open. Several studies reported an association between these two diseases (3, 16, 17, 22), while others did not find any significant relationship (27, 28). In 2013, Chui et al. demonstrated that autoimmune thyreopathy and chronically present phlogistic infiltrates could lead to the alteration of the follicular epithelium of the thyroid, which in fact could be responsible for the dysplastic transformation of the follicular epithelium and to the formation of so-called “zones of follicular dysplasia”. This is a preneoplastic lesion that can evolve into papillary thyroid cancer (3, 29). Particular attention is given in the literature to the research of novel prognostic markers in thyroid cancer and the possible role of HT as a risk factor in the development of DTC; therefore, its role as a favorable prognosis factor continues to keep the discussion alive among the experts (3, 15–17, 20, 22, 30–36).

The aim of our study was to examine the relationship between HT and DTC, evaluate whether HT could be an independent risk factor for DTC, and assess the characteristics of DTC with concomitant HT in terms of risk of structural disease recurrence and disease-free survival when compared to DTC without concomitant HT.

In our study, the frequency of concomitant HT with DTC on surgical specimens was significantly higher than in non-DTC patients: approximately 50.1% of DTC patients were diagnosed with a concomitant HT, while only 38.6% of non-DTC patients were found to have a concomitant HT diagnosis on pathological examination (p < 0.001). These results are concordant with the most recent literature, as other studies before ours have pointed out an association between HT and DTC (3, 16, 17, 20, 22).

Notably, our study found no difference in terms of preoperative administration of substitutive levothyroxine therapy between the DTC and non-DTC groups; furthermore, DTC patients were significantly younger than non-DTC patients. This difference could be explained by a slightly earlier diagnosis of DTC with HT, as those patients are likely more prone to close follow-up, as already pointed out by Battistella et al. (22). It should also be emphasized that our sample is represented entirely by surgical patients; thus, a selection bias could be present regarding age: it is possible that the age difference is due to earlier intervention in DTCs than in patients with benign disease.

In the multivariable analysis we found that the presence of HT was an independent risk factor for developing DTC (OR 1.778, 95% CI 1.333 to 2.372, p < 0.001). These results are similar to those published by Apostolou et al. in their 2021 manuscript, which shows an OR for thyroid cancer development of 2.31 when concomitant HT was diagnosed (95% CI 1.85-2.89, p < 0.001) (37).

Many authors have reported a considerably better prognosis of DTC when HT is also present; however, these data are not uniform in the various studies, as some report a greater presence of multifocal carcinomas and a similar recurrence rate to non-HT tumors (20, 21), while others show a greater presence of microcarcinomas, with a smaller average tumor size and a lower incidence of lymph node metastases and extrathyroidal extension, leading to significantly better disease-free survival for DTCs with concomitant HT than for those without HT (16, 22, 23, 38).

The 2015 ATA guidelines proposed a classification of risk of structural disease recurrence for DTCs, based on histological features of aggressiveness, such as extrathyroidal extension, vascular invasion, nodal metastasis, multifocality and aggressive variants. According to that, DTCs could be divided into three categories: low risk, intermediate risk and high risk, which identify a progressive risk of disease recurrence (26). As reported in table 4, we stratified DTC patients based on this classification. However, no statistically significant difference was found between HT and non-HT carcinomas.

We subsequently proceeded to analyze tumor characteristics with and without the coexistence of HT in surgical specimens, HT-DTC, and non-HT-DTC groups respectively. The results are shown in **Table 3**. A clear female predominance was present in the HT-DTC group, as widely foreseeable given the presence of HT. A statistically significant difference was found in tumor multifocality, which was present in 40.5% of HT-DTC, while only 29.6% of non-HT-DTC tumors were classified as multifocal (p=0.02). Our results are concordant with the studies in the literature, which report a slightly higher presence of multifocal thyroid cancers when HT is present (20, 21). Furthermore, HT-DTC tumor size was significantly smaller than non-HT-DTC, 13.7 ± 11.9 mm vs 17.6 ± 16.5 mm (p=0.007). According to that, 44% of HT-DTC was a microcarcinoma, compared to only 34.2% of the non-HT-DTC group (p=0.05). These results are similar to those reported by Battistella et al., Uhliarova et al., and Liang et al. (16, 20, 22) and support the thesis that DTCs with concomitant HT exhibit less aggressive histological features, although our study did not report statistically significant differences in terms of lymph node metastases and extrathyroidal extension in HT carcinomas, while some authors report a lower incidence of these aggressive features in HT-carcinomas, as pointed out before in this paragraph.

Globally, our overall survival was 99.5%, and our recurrence rate was 3%, with a mean time between surgery and diagnosis of recurrence of 50.5 ± 13.8 months. No statistically significant difference between HT-DTC and non-HT-DTC groups was found regarding recurrence rate and overall survival. Our study found a significant difference in terms of post-operative administration of RAI therapy, which was given in 63.7% in the HT-DTC group and in 69% in the non-HT-DTC group (p=0.04). These results are in line with those reported in the literature in the study of Lau et al., where the authors found that HT patients tend to have fewer cycles of RAI therapy and correspondingly have excellent response to treatment. Although our study found no influence of HT on recurrence and survival rates, this is probably due to the relatively small sample size; in the literature, a recent meta-analysis performed by Xu et al. showed a better prognosis of HT carcinomas than non-HT carcinomas, with a reduction both in mortality rate and recurrence rate (21).

Our study has some limitations. First, it is a retrospective study, even if we want to emphasize that data were extracted from a prospectively maintained database. The second limitation, as previously reported, is that our sample is based only on patients already submitted to thyroid surgery; therefore, it may not be fully representative of the general population. Finally, the real incidence of disease recurrence could be underestimated in our study, considering that the mean follow-up is 50.5 months, that these kinds of tumors are generally indolent, and that recurrences can appear up to 10 years after surgery.

## Conclusion

In conclusion, the relationship between HT and DTC is far from explained. HT is frequently coexistent with DTCs in surgical specimens, and in our study, HT was found to be an independent risk factor for developing DTC. Furthermore, DTCs developed with concomitant HT appear to be smaller tumors than DTCs developed without HT, and even if our study finds no difference in risk of structural disease recurrence and recurrence rates between HT-DTC and non-HT-DTC, data in the literature suggest that DTCs with concomitant HT are characterized by less aggressive histological features and better prognosis than those without HT. Given our data, and considering those already present in the literature, we think that a strict follow-up of HT patients is advisable.

## Data availability statement

The raw data supporting the conclusions of this article will be made available by the authors, without undue reservation.

## Ethics statement

The studies involving human participants were reviewed and approved by Independent Ethics Committee, A.O.U. Cagliari. The patients/participants provided their written informed consent to participate in this study.

## Author contributions

FC: study conception and design, acquisition of data, analysis and interpretation of data, drafting of the article, final approval of the version to be submitted. GC: acquisition of data, drafting of the article, final approval of the version to be submitted. ML: analysis and interpretation of data, final approval of the version to be submitted. EL: analysis and interpretation of data, revision of the article for important content, final approval of the version to be submitted. MB: acquisition of data, analysis and interpretation of data, final approval of the version to be submitted. FB: analysis and interpretation of data, revision of the article for important content, final approval of the version to be submitted. FM: study conception and design, acquisition of data, drafting of the article, final approval of the version to be submitted. All authors contributed to the article and approved the submitted version.

## Funding

This research did not receive any specific grant from funding agencies in the public, commercial, or not-for-profit sectors.

## Acknowledgments

We thank Mohamad Abbas, Valerio Argiolas, Maria Boe, Francesco Casti and Silvia Puddu, our residents, for their precious collaboration in data collection.

## Conflict of interest

The authors declare that the research was conducted in the absence of any commercial or financial relationships that could be construed as a potential conflict of interest.

## Publisher’s note

All claims expressed in this article are solely those of the authors and do not necessarily represent those of their affiliated organizations, or those of the publisher, the editors and the reviewers. Any product that may be evaluated in this article, or claim that may be made by its manufacturer, is not guaranteed or endorsed by the publisher.
